# Diminished HIV Infection of Target CD4+ T Cells in a Toll-Like Receptor 4 Stimulated *in vitro* Model

**DOI:** 10.3389/fimmu.2019.01705

**Published:** 2019-07-23

**Authors:** Ross Cromarty, Alex Sigal, Lenine J. P. Liebenberg, Lyle R. McKinnon, Salim S. Abdool Karim, Jo-Ann S. Passmore, Derseree Archary

**Affiliations:** ^1^Centre for the AIDS Programme of Research in South Africa, Nelson Mandela School of Medicine, University of KwaZulu-Natal, Durban, South Africa; ^2^Africa Health Research Institute, University of KwaZulu-Natal, Durban, South Africa; ^3^Max Planck Institute for Infection Biology, Berlin, Germany; ^4^Department of Medical Microbiology, University of KwaZulu-Natal, Durban, South Africa; ^5^Department of Medical Microbiology and Infectious Diseases, University of Manitoba, Winnipeg, MB, Canada; ^6^Department of Epidemiology, Mailman School of Public Health, Columbia University, New York, NY, United States; ^7^Medical School, Institute of Infectious Diseases and Molecular Medicine, University of Cape Town, Cape Town, South Africa; ^8^National Health Laboratory Service, Cape Town, South Africa

**Keywords:** Toll-like receptors, inflammation, immune activation, HIV, cytokines, innate antiviral immunity

## Abstract

Genital inflammation is associated with increased HIV acquisition risk. Induction of an inflammatory response can occur through the recognition of pathogenic or commensal microbes by Toll-like receptors (TLRs) on various immune cells. We used a *in vitro* peripheral blood mononuclear cell (PBMC) system to understand the contribution of TLR stimulation in inducing inflammation and the activation of target T cells, and its effect on HIV susceptibility. PBMCs were stimulated with TLR agonists LPS (TLR4), R848 (TLR7/8), and Pam3CSK4 (TLR1/2), and then infected with HIV NL4-3 AD8. Multiplexed ELISA was used to measure 28 cytokines in cell culture supernatants. Flow cytometry was used to measure the activation state (CD38 and HLA-DR), and CCR5 expression on CD4+ and CD8+ T cells. Although TLR agonists induced higher cytokine and chemokine secretion, they did not significantly activate CD4+ and CD8+ T cells and showed decreased CCR5 expression relative to the unstimulated control. Despite several classes of inflammatory cytokines and chemokines being upregulated by TLR agonists, CD4+ T cells were significantly less infectable by HIV after TLR4-stimulation than the unstimulated control. These data demonstrate that the inflammatory effects that occur in the presence TLR agonist stimulations do not necessarily translate to the activation of T cells. Most importantly, the finding that TLR4-stimulation reduces rather than increases susceptibility of CD4+ T cells to HIV infection in this *in vitro* system strongly suggests that the increased chemokine and possible antiviral factor expression induced by these TLR agonists play a powerful although complex role in determining HIV infection risk.

## Introduction

HIV and AIDS is a global epidemic that affects approximately 37 million people worldwide, with an additional 1.8 million new HIV infections documented in 2017 (1). Sub-Saharan Africa bears more than half of the global HIV burden, with young adolescent women (aged 15–24 years) twice as likely to be living with HIV compared to men in this region (1). Furthermore, 75% of new HIV infections among adolescents (15–19 years) are in girls (1). Specifically, South Africa accounts for 19% of HIV infected people globally, 15% of new HIV infections annually and 11% of AIDS related deaths worldwide (2). The inception of antiretroviral therapy (ART) has dramatically reduced the risk of HIV related morbidity and mortality, and has transformed the epidemic into a manageable chronic disease (3). Strategies to prevent infection are crucial for control and eventual eradication of the HIV epidemic. Various pre-exposure prophylaxis (PrEP) strategies such as oral tablets, microbicides or intra-vaginal rings containing anti-retroviral drugs, have been proposed with various levels of success.

Many social, behavioral and biological factors undermine the efficacy of these prevention strategies (4–6). One important biological factor is female genital tract inflammation. Genital inflammation, defined by the increase in inflammatory and chemotactic cytokines such as IL-1α, IL-1β, TNF-α, MIP-1α, MIP-1β, IP-10, and IL-8, among others, has been associated with an increased risk of HIV acquisition (7–9). One of the mechanisms implicated is that inflammation increases HIV risk by causing activation of HIV target cells (CD4+ T cells), thereby priming the cells for HIV infection (10). Inflammation also leads to increased recruitment of these activated target CD4+ T cells to the environment where infection occurs (11). Additionally, T cell activation profiles in the blood predicted those in the genital tract (12), suggesting that these activation profiles in the blood could be a surrogate indication of activation in the genital tract with subsequent increased risk for HIV. Lastly genital inflammation leads to the disruption of the mucosal barrier, which is not only more permissive to viral translocation (11) but also facilitates infection with less infectious virions (13). Furthermore, genital inflammation has been shown to reduce the protective effect of TFV 1% gel as a vaginal microbicide in the CAPRISA 004 trial (6, 14). Additionally, a dysbiotic microbiome or bacterial vaginosis (BV), which is often associated with genital inflammation (15, 16), also undermined the efficacy of the 1% TFV gel microbicide (17). The reduced efficacy was attributed to the direct metabolism of TFV by *Gardnerella vaginalis* (17), a microbe which is often associated with BV (18).

Inflammation is the natural biological response for protection against invading pathogens and damaged tissue. Inflammation can be broadly defined into three stages; recognition and release, activation and recruitment, and resolution and repair (19). The causes of genital inflammation have yet to be fully understood, however, sexually transmitted infections (STI) and a dysbiotic microbiome have been implicated (9, 15, 20). The mechanisms underlying the induction of inflammation by these two factors likely involve the recognition of pathogen associated molecular patterns (PAMPs) by Toll-Like Receptors (TLRs) (21), a cardinal pathway for the induction of an immune response (22). Various TLRs are able to recognize a broad range of antigens, from bacterial wall proteins to various types of bacterial and viral genetic products (23), and each TLR initiates a distinct signaling cascade for the induction of innate immune responses (24, 25). TLRs are expressed to various degrees on most immune cells, with innate antigen presenting cells expressing the widest range (23). Common PAMPs that are known to have significant immunological effects include lipopolysaccharide (LPS) recognized by TLR4 (26, 27), single stranded RNA (ssRNA) recognized by TLR7/8 (28, 29) and bacterial lipopeptides recognized by TLR1/2 (30, 31). TLR-stimulation of mouse splenocytes with R848 (TLR7/8 agonist) increased IL-1α, IL-2 and IL-6 expression, while LPS increased IL-1α, IL-2 and IL-4 expression (32). Additionally, Wang *et al*. demonstrated that human peripheral blood mononuclear cells (PBMCs) stimulated with LPS induced the production of IL-1β, TNF-α, IL-6, and IL-22 (32). Similarly in a study investigating Th17 cell induction in human PBMCs, the TLR agonists R848 and LPS elicited production of IP-10, IL-6, MCP-1, IL-8, MIP-1α, and RANTES, while R848 further induced IL-12(p40), IL-1β, and TNF-α production (33). Furthermore, in the context of vaccine induced immunity, very similar cytokine responses from human monocyte-derived-DC's (MDDCs) and monocyte-derived-macrophages (MDMs) were found with vaccine adjuvants R848 and the TLR4 agonist Glucopyranosyl Lipid Adjuvant (GLA) (34). A strong chemokine response was observed with high expression of MIP-1α, MIP-1β, RANTES, and IP-10, while the pro-inflammatory cytokine response was less pronounced, with lower expression of IL-1α, IL-1β, and TNF-α compared to the chemokines (34).

TLR agonists have been shown to induce potent inflammatory responses and genital inflammation has been associated with the increased risk of HIV acquisition. Therefore, we sought to recapitulate some of the findings from genital inflammation studies using an *in vitro* PBMC system to understand the contribution of TLR-mediated inflammatory response to the activation and HIV infection of target CD4+ T cells.

## Materials and Methods

### Ethics Statement

This study was carried out in accordance with the recommendations of the University of KwaZulu-Natal (UKZN) Biomedical Research Ethics Committee (BREC). All subjects gave written informed consent in accordance with the Declaration of Helsinki. The protocol was approved by the UKZN BREC (BE433/14).

### Cell Culture Media

C10 media was used for all cell culture experiments. C10 media consisted of RPMI 1640 with L-glutamine (Lonza, Basel, Switzerland) containing 10% FCS (non-Hi FCS; Highveld Biological, JHB, SA), 2% L-glutamine, 1% HEPES, 1% NaPy, 1% NEAA (all from Lonza, Basel, Switzerland). Media was sterile filtered through the Filtermax 500 ml (Techno Plastic Products, Trasadingen, Switzerland). IL-2 (PeproTech, Rocky Hill, NJ, USA), was added to C10 media prior to use at a final concentration of 0.01 μg/ml.

### Stimulants and HIV Strain

The TLR agonists LPS (TLR4), R848 (TLR7/8), and Pam3CSK4 (TLR1/2) (all from Invivogen, San Diego, CA, USA) concentrations were previously optimized in TLR titration experiments. As no significant differences were observed in HIV infections (Supplementary Figure 1) or cytokine profiles (Supplementary Figures 2–4) between the TLR concentrations, a final concentration of 2 μg/ml was used. Phytohaemagglutinin (PHA) (Sigma-Aldrich, St. Louis, MO, USA), used as the positive control at a final concentration of 10μg/ml. Unstimulated PBMCs were used as the negative control. The CCR5-tropic HIV-1 NL4-3 AD8 (35) (a gift from Dr. Alex Sigal), was used at a working dilution of 1:20, which corresponded to an MOI of 0.9, which had been previously optimized (data not shown). PHA and unstimulated uninfected conditions were used as controls for HIV.

### Antibodies

Cellular activation was assessed by measurement of HLA-DR and CD38, similar to previous studies (12, 36, 37). Staining for flow cytometry was performed both extracellularly and intracellularly. The extracellular staining cocktail consisted of LIVE/DEAD Amcyan fixable dye (Thermo Fisher Scientific, Waltham, MA, USA), anti-CD3-APC-H7, anti-CD4-BV605, anti-CD8-BV655, anti-CD14-Pacific blue (all from BD Biosciences, Franklin Lakes, NJ, USA), and anti-CD19-pacific blue (Biolegend, San Diego, CA, USA). The intracellular staining cocktail consisted of anti-CCR5-APC, anti-HLA-DR-PerCP-CY5.5 (all from BD Biosciences, Franklin Lakes, NJ, USA), anti-CD38-PE-CY7 (Biolegend, San Diego, CA, USA) and anti-p24-FITC (Beckman Coulter, Brea, CA, USA). PBMCs were collected at two time-points: day 3 (48 h post stimulation and prior to HIV infection) and day 5 (48 h post infection).

### Cell Culture

The cell culture and HIV infection protocol used in this study was adapted from previous studies (38, 39). Blood was collected from 5 healthy volunteer donors and PBMCs were isolated by density gradient centrifugation. PBMCs were resuspended to 1 × 10^6^ cells/ml in C10 media and plated into cell culture plates. PBMCs were left either unstimulated (as a negative control) or stimulated immediately after plating with TLR agonists or PHA, which was used as a positive control. Following stimulation, the PBMCs were cultured at 37°C 5% CO_2_ for 48 h. Following this incubation, the contents of each well was collected into 15 ml falcon tubes and an aliquot of 500 μl was removed for multiplexed ELISA (culture supernatants) and flow cytometry (PBMCs) for the day 3 time-point (post stimulation, prior to HIV infection). The 15 ml falcon tubes were centrifuged, supernatants were discarded, and media replacement was performed with fresh C10 media. PBMCs were plated at 1 × 10^6^ cells/ml into 24-well cell culture plates, no further TLR or PHA stimulations were performed. Subsequently, HIV infection was done by the addition of 250 μl at a 1:20 dilution of the HIV-1 NL4-3 AD8 viral stocks at a MOI of 0.9. PHA and unstimulated uninfected wells were treated with 250 μl C10 media. Plates were incubated at 37°C 5% CO_2_ for 48 h, whereupon multiplexed ELISA (culture supernatants) and flow cytometry (PBMCs) was performed for the day 5 time-point (post HIV infection).

### Flow Cytometry

PBMCs were centrifuged at 3,500 rpm for 5 min to pellet the cells and remove soluble HIV, and the cell culture supernatants were stored at −80°C for cytokine quantification. PBMCs were washed with sterile PBS supplemented with 2% FCS and then stained with 100 μl extracellular staining cocktail, fixed, and then stained with 100 μl intracellular staining cocktail. Data was acquired by flow cytometry on a BD LSR Fortessa (BD Biosciences, Franklin Lakes, NJ, USA). Data analysis was performed using FlowJo v10.4.1 software (Tree Star, Ashland, OR, USA), according to the gating strategy (Supplementary Figure 5). For the purpose of this study we reported on four activation phenotypes and defined these as the following; The CD38+HLA-DR+ phenotype was defined as hyper-activated, the CD38+HLA-DR- and CD38-HLA-DR+ phenotypes were defined as intermediately activated, and the CD38-HLA-DR- phenotype was defined as resting or not activated.

### Cytokine Quantification

The concentrations of 28 cytokines were assessed from cell culture supernatants using the Bio-Plex Pro Human Cytokine Group I 27-Plex Panel (Bio-Rad Laboratories, Hercules, CA, USA) and the Magnetic Luminex® Assay IL-1α Singleplex Kit (Research and Diagnostic (R&D) systems Inc., Minneapolis, Minnesota, USA) as per manufacturer's instructions. Data was acquired on the Bio-Plex® 200 system (Bio-Rad Laboratories, Hercules, CA, USA). Optimization of standard curves were performed automatically using the Bio-Plex manager software version 6.1 (Bio-Rad Laboratories, Hercules, CA, USA). Values with coefficients of variation <20% and with observed recoveries between 70 and 130% were considered reliable. Values that were below the detectable limit were assigned half of the lowest limit of detection value (LLOD), while values that were above the detectable limit were assigned double the highest limited of detection (HLOD) value.

### Statistical Analysis

Statistical analyses and graphical representation of data was performed using the GraphPad Prism version 7.02 software for windows (GraphPad Software, La Jolla, CA, USA). For comparisons of cellular activation markers CD38, HLA-DR on CD4+ and CD8+ T cells, between stimulated conditions and the unstimulated control, a repeated measures two-way ANOVA with a Dunnett's multiple comparisons test was performed. Similarly, for CCR5 and cytokine comparisons, an ordinary one-way ANOVA with Dunnett's multiple comparison test was performed. Cellular activation results are displayed as mean percentage (%) ± standard deviation (SD) of CD4+ or CD8+ T cells. Cytokine data was normalized by Log_10_ transformation and is displayed as mean concentration (Log_10_ pg/ml) ± standard deviation (SD). To understand the effect of various TLR agonists on cytokine expression, heat maps were generated by performing a single linkage hierarchical cluster analysis using R version 3.3.3 statistical software (R Foundation for Statistical Computing, Vienna, Austria). Radial spider plots were created using Microsoft Excel© 2013 software (Microsoft Corporation, Redmond, WA, USA).

## Results

### TLR Stimulation Did Not Result in the Activation of CD4+ T Cells

**Figure 1:**
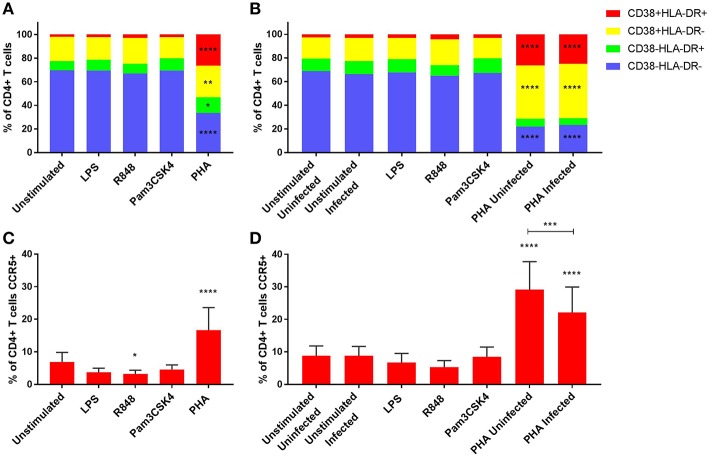
Activation profiles **(A,B)** and CCR5 expression **(C,D)** of CD4+ T cells on day 3 prior to HIV infection **(A,C)** and day 5 post HIV infection **(B,D)**. PHA was used at a final concentration of 10 μg/ml. TLR agonists were used at a final concentration of 2 μg/ml. A repeated measures two-way ANOVA with Dunnett's multiple comparisons test was used for immune activation, and an ordinary one-way ANOVA with a Dunnett's multiple comparisons test for CCR5 expression. Significance is displayed as ^*^p < 0.05, ^**^p < 0.01, ^***^p < 0.001, ^****^p ≤ 0.0001 compared to the unstimulated/unstimulated infected control, unless otherwise shown. Sample size, *n* = 5, 4 donors run in quadruplicate, 1 donor run in duplicate.

Minimal cytotoxic effects were observed with TLR stimulation, apart from R848 where a significant reduction in cell viability was observed (Supplementary Figure 6). As highly activated CD4+ T cells have been shown to be preferentially infected (10), we determined how TLR stimulation impacted on the expression of the activation markers HLA-DR and CD38 on CD4+ T cells. TLR stimulation did not induce significant CD4+ T cell activation compared to the unstimulated control (*p* > 0.05) at day 3 (post stimulation, prior to HIV infection) or day 5 (post infection) (Figure 1). However, when PBMCs were stimulated with the mitogen PHA, distinct increased cellular activation was observed, with all three activation phenotypes significantly increased compared to the unstimulated control on day 3 (*p* < 0.05). Similarly, on day 5 and irrespective of infection status, PHA induced significantly elevated expressions of CD38+HLA-DR+ and CD38+HLA-DR-, but not CD38-HLA-DR+ on CD4+ T cells compared to the unstimulated infected control (*p* ≤ 0.0001) (Figure 1B). Representative dot plots of flow cytometric data are shown in Supplementary Figure 7. Relevant mean ± SD for data depicted in Figure 1 are listed in Supplementary Table 1.

### TLR Activation Downregulated CCR5 Expression on CD4+ T Cells

Since CCR5 is a co-receptor for R5-tropic HIV infection, we determined how TLR activation impacted the expression of CCR5 by CD4+ T cells. R848 stimulation significantly lowered CCR5 expression (3.2 ± 1.2% of CD4+ T cells) compared to the unstimulated control (6.9 ± 2.9% of CD4+ T cells) (*p* < 0.05), while PHA significantly increased the CCR5 expression (16.6 ± 6.9% of CD4+ T cells) (*p* ≤ 0.0001) at day 3 (Figure 1C). Of note, CCR5 expression was significantly lower in PHA-stimulated infected condition by day 5 (22.1 ± 7.9% of CD4+ T cells) compared to the PHA-stimulated but uninfected condition (29.1 ± 8.6% of CD4+ T cells) (*p* = 0.0003), although both conditions had significantly higher CCR5 expression than the unstimulated but HIV-infected control (8.8 ± 2.9% of CD4+ T cells) (Figure 1D). Representative dot plots of flow cytometric data are shown in Supplementary Figure 7.

### R848 (TLR7/8) Induced Activation of CD8+ T Cells

**Figure 2:**
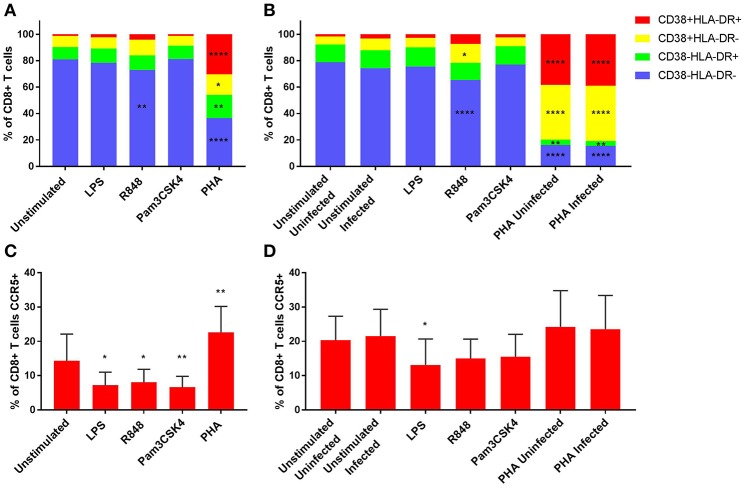
Activation profiles **(A,B)** and CCR5 expression **(C,D)** of CD8+ T cells on day 3 prior to HIV infection **(A,C)** and day 5 post HIV infection **(B,D)**. PHA was used at a final concentration of 10 μg/ml. TLR agonists were used at a final concentration of 2 μg/ml. A repeated measures two-way ANOVA with Dunnett's multiple comparisons test was used for immune activation, and an ordinary one-way ANOVA with a Dunnett's multiple comparisons test for CCR5 expression. Significance is displayed as ^*^p < 0.05, ^**^p < 0.01, ^****^p ≤ 0.0001 compared to the unstimulated/unstimulated infected control, unless otherwise shown. Sample size, *n* = 5, 4 donors run in quadruplicate, 1 donor run in duplicate.

As CD8+ T cells are important effector cells and are crucial in viral control, we sought to assess the effect of TLR activation on CD8+ T cells. Similar findings were observed in the CD8+ and CD4+ T cells, with no significant activation observed with LPS or Pam3CSK4 stimulations compared to the unstimulated control at day 3 (Figure 2A). While there was a significant reduction of inactivated (CD38-HLA-DR-) CD8+ T cells with R848-stimulation compared to the unstimulated control (*p* < 0.01), this did not translate to a significant increase in any of the activated phenotypes (Figure 2A). PHA induced significant cellular activation, with all three activation phenotypes significantly increased compared to the unstimulated control (*p* < 0.05) on day 3 (Figure 2A). On day 5, only R848 significantly increased the frequency of CD8+ T cells expressing CD38+HLA-DR- (*p* < 0.05), compared to unstimulated cells (*p* ≤ 0.0001) (Figure 2B). PHA, irrespective of infection status, maintained elevated levels of the activation phenotypes CD38+HLA-DR+ and CD38+HLA-DR- (*p* ≤ 0.0001) compared to the unstimulated infected control (Figure 2B). Representative dot plots of flow cytometric data are shown in Supplementary Figure 8. Relevant mean ± SD for data depicted in Figure 2 are listed in Supplementary Table 2.

### TLR-Mediated Reduction of CCR5 Expression on CD8+ T Cells Is Restored Over Time

CCR5 expression by CD8+ T cells was significantly lower than the unstimulated control (14.3 ± 7.8% of CD8+ T cells) with LPS (7.2 ± 3.8% of CD8+ T cells) (*p* < 0.05), R848 (8.1 ± 3.8% of CD8+ T cells) (*p* < 0.05) or Pam3CSK4 (6.7 ± 3.2% of CD8+ T cells) (*p* < 0.01) on day 3 (Figure 2C). Conversely, significantly elevated CCR5 expression on CD8+ T cells was observed with PHA (22.6 ± 7.6% of CD8+ T cells) compared to the unstimulated control (*p* < 0.01) on day 3. Only LPS (13.1 ± 7.6% of CD8+ T cells) maintained significantly lower CCR5 expression on CD8+ T cells than the unstimulated infected control (21.6 ± 7.8% of CD8+ T cells) on day 5 (*p* < 0.05) (Figure 2D). Representative dot plots of flow cytometric data are shown in Supplementary Figure 8.

### LPS (TLR4) and R848 (TLR7/8) Induced Strong Inflammatory Cytokine Responses

**Figure 3:**
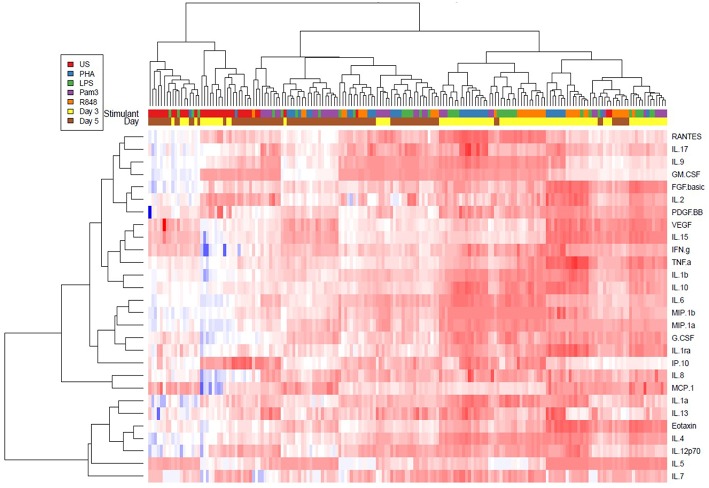
Unsupervised hierarchical cluster heat map analysis of 28 cytokines measured in cell culture supernatants on day 3 (yellow) and day 5 (brown) from the unstimulated (red), PHA (blue), LPS (green), Pam3CSK4 (purple), or R848 (orange) conditions. PHA was used at a final concentration of 10 μg/ml. TLR agonists were used at a final concentration of 2 μg/ml. Sample size, *n* = 5, 4 donors run in quadruplicate, 1 donor run in duplicate.

Unsupervised hierarchical clustering analysis and Radial spider plots were used to evaluate cytokine production by PBMCs in response to stimulation with various TLR agonists (Figure 3 and Supplementary Figures 9, 10, respectively). Pam3CSK4 (TLR1/2) did not induce much cytokine production and tended to cluster closely with the unstimulated conditions, while LPS, R848, and PHA tended to cluster together, with similarly elevated inflammatory cytokine profiles. Cytokine induction by these TLR agonists appeared to be higher at day 3 than day 5 (Figure 3).

### TLR4 and TLR7/8 Activation Induced the Greatest Inflammatory Profile, With TLR7/8 Activation Maintaining Inflammatory Cytokine Profile

**Figure 4:**
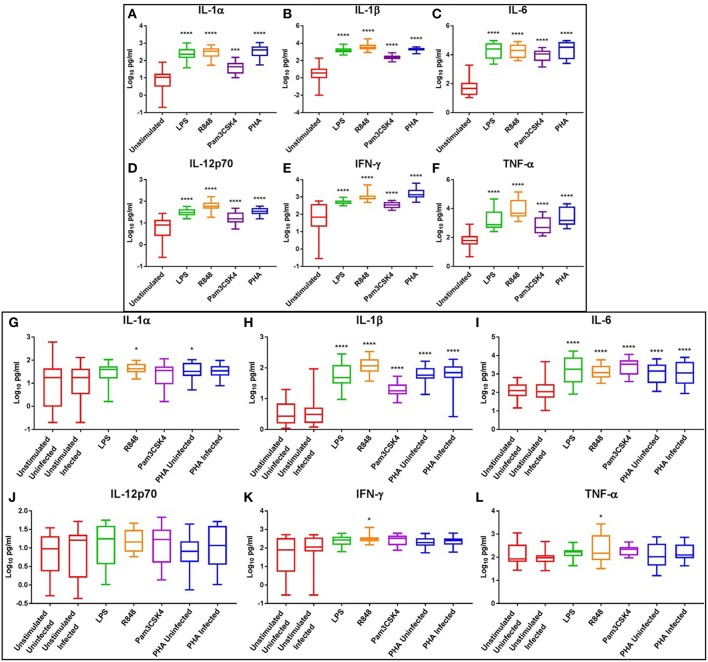
Box and Whisker plots showing mean ± SD Log10 concentrations of pro-inflammatory cytokines IL-1α **(A,G)**, IL-1β **(B,H)**, IL-6 **(C,I)**, IL-12p70 **(D,J)**, IFN-γ **(E,K)**, and TNF-α **(F,L)** from unstimulated (red), LPS (green), R848 (orange), Pam3CSK4 (purple), and PHA (blue) conditions on day 3 prior to HIV infection (top box: **A–F**) and day 5 post HIV infection (bottom box: **G–L**). TLR agonists were used at a final concentration of 2 μg/ml. PHA was used at a final concentration of 10 μg/ml. All TLR stimulation conditions were infected. An ordinary one-way ANOVA with a Dunnett's multiple comparisons test was performed. Significance is displayed as ^*^p < 0.05, ^***^p < 0.001, ^****^p ≤ 0.0001 compared to the unstimulated control. Sample size, *n* = 5, 4 donors run in quadruplicate, 1 donor run in duplicate.

As previous studies have shown that genital inflammation, defined by increased concentrations of a subset of 12 inflammatory cytokines and chemokines (including IL-1α, IL-1β, IL-6, IL-7, IL-8, IL-10, TNF-α, IP-10, MIP-1α, MIP-1β, MCP-1, and GM-CSF), predicted >3-fold increased risk for HIV acquisition (7), we sought to focus further analysis on the effect of TLR activation on these cytokines and 4 others (IL-12p70, IFN-γ, RANTES, and IL-17) that have crucial immunological roles. Both LPS and R848 induced significant production of the pro-inflammatory cytokines IL-1α, IL-1β, IL-6, IL-12p70, IFN-γ, and TNF-α at day 3 (*p* ≤ 0.0001 and *p* ≤ 0.0001, respectively) compared to the unstimulated control (Figures 4A–F). Pam3CSK4 also elicited significantly elevated pro-inflammatory cytokines compared to the unstimulated control (*p* < 0.001), however these levels were generally lower than those observed with LPS or R848 (Figures 4A–F).

Although cytokine induction was declining by day 5, pro-inflammatory cytokines IL-1β and IL-6 remained significantly elevated in the LPS (*p* ≤ 0.0001), R848 (*p* ≤ 0.0001), Pam3CSK4 (*p* ≤ 0.0001), and PHA conditions (*p* ≤ 0.0001) compared to the unstimulated HIV-infected control (Figures 4H,I). IL-1α was significantly elevated in the R848 and PHA uninfected conditions (*p* < 0.05) (Figure 4G), while IFN-γ and TNF-α were significantly elevated only in the R848 condition (*p* < 0.05) (Figures 4K,L). Relevant mean ± SD for data depicted in Figure 4 are listed in Supplementary Table 3.

### Potent Chemokine Response to TLR Activation, With Concomitant Downregulation of IP-10

**Figure 5:**
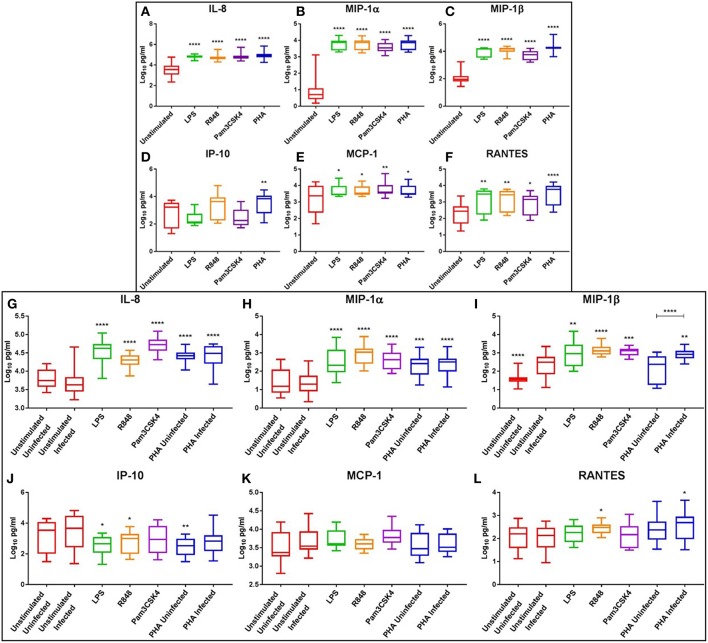
Box and Whisker plots showing mean ± SD Log10 concentrations of chemotactic cytokines IL-8 **(A,G)**, MIP-1α **(B,H)**, MIP-1β **(C,I)**, IP-10 **(D,J)**, MCP-1 **(E,K)**, and RANTES **(F,L)** from unstimulated (red), LPS (green), R848 (orange), Pam3CSK4 (purple), and PHA (blue) conditions on day 3 prior to HIV infection (top box: **A–F**) and day 5 post HIV infection (bottom box: **G–L**). TLR agonists were used at a final concentration of 2 μg/ml. PHA was used at a final concentration of 10 μg/ml. All TLR stimulation conditions were infected. An ordinary one-way ANOVA with a Dunnett's multiple comparisons test was performed. Significance displayed as ^*^p < 0.05, ^**^p < 0.01, ^***^p < 0.001, ^****^p ≤ 0.0001 compared to the unstimulated control. Sample size, *n* = 5, 4 donors run in quadruplicate, 1 donor run in duplicate.

At day 3, PHA stimulation or TLR activation with LPS, R848 or Pam3CSK4 significantly increased the levels of chemotactic cytokines IL-8 (*p* ≤ 0.0001), MIP-1α (*p* ≤ 0.0001), MIP-1β (*p* ≤ 0.0001), MCP-1 (*p* < 0.05), and RANTES (*p* < 0.05) compared to the unstimulated control (Figures 5A–C,E,F). Additionally, IP-10 was significantly increased with PHA stimulation compared to the unstimulated control (*p* < 0.01) (Figure 5D). R848 appeared to be a more potent inducer of IP-10 than either LPS or Pam3CSK4 (Figure 5D).

At day 5, the chemokines IL-8 and MIP-1α remained significantly elevated in the LPS (*p* ≤ 0.0001), R848 (*p* ≤ 0.0001), Pam3CSK4 (*p* ≤ 0.0001) and PHA conditions (*p* < 0.001) compared to the unstimulated infected control (Figures 5G,H). TLR activation with LPS (*p* < 0.01), R848 (*p* ≤ 0.0001), or Pam3CSK4 (*p* < 0.001) significantly increased MIP-1β compared to the unstimulated infected control (Figure 5I). The PHA infected, but not the uninfected, condition had significantly increased MIP-1β compared to the unstimulated infected control (*p* < 0.01) (Figure 5I). Interestingly, with regards to MIP-1β, the unstimulated and PHA infected conditions had significantly greater concentrations than the matched uninfected conditions (*p* ≤ 0.0001) (Figure 5I). Concentrations of RANTES were significantly elevated in the R848 and PHA infected conditions compared to the unstimulated control (*p* < 0.05) (Figure 5L).

Conversely, IP-10 concentrations were significantly reduced in the LPS (*p* < 0.05), R848 (*p* < 0.05) and the PHA uninfected (*p* < 0.01) conditions compared to the unstimulated infected control (Figure 5J). Relevant mean ± SD for data depicted in Figure 5 are listed in Supplementary Table 4.

### Potent Induction of IL-17 Response With TLR Agonists LPS (TLR4), R848 (TLR7/8) and Pam3CSK4 (TLR1/2)

**Figure 6:**
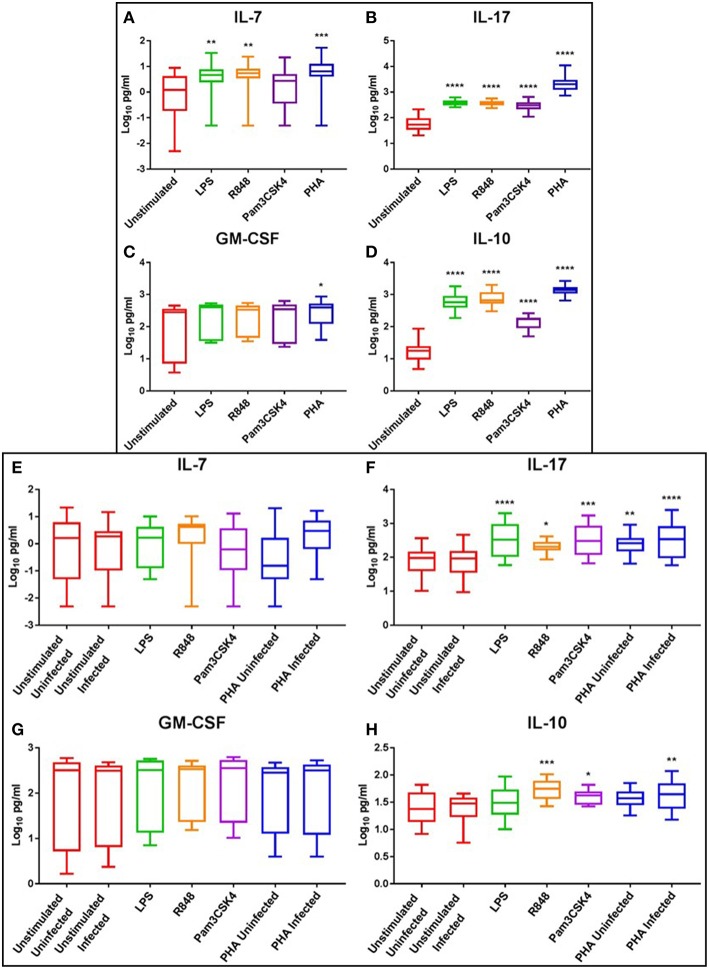
Box and Whisker plots showing mean ± SD Log10 concentrations of haematopoietic cytokines IL-7 **(A,E)** and IL-17 **(B,F)**, the growth factor GM-CSF **(C,G)**, and the anti-inflammatory cytokine IL-10 **(D,H)** from unstimulated (red), LPS (green), R848 (orange), Pam3CSK4 (purple), and PHA (blue) conditions on day 3 prior to HIV infection (top box: **A–D**) and day 5 post HIV infection (bottom box: **E–H**). TLR agonists were used at a final concentration of 2 μg/ml. PHA was used at a final concentration of 10 μg/ml. All TLR stimulation conditions were infected. An ordinary one-way ANOVA with a Dunnett's multiple comparisons test was performed. Significance is displayed as ^*^p < 0.05, ^**^p < 0.01, ^***^p < 0.001, ^****^p ≤ 0.0001 compared to the unstimulated control. Sample size, *n* = 5, 4 donors run in quadruplicate, 1 donor run in duplicate.

At day 3, the haematopoietic IL-7 and IL-17 were significantly elevated following LPS (*p* < 0.01), R848 (*p* < 0.01), and PHA stimulation (*p* < 0.001) compared to the unstimulated controls, while only IL-17 was significantly elevated with Pam3CSK4 (*p* ≤ 0.0001) (Figures 6A,B). Interestingly, IL-17 increased in a dose-dependent manner with R848 stimulation, with this effect more prominent at day 3 than day 5 (Supplementary Figure 11). PHA, but not TLR activation, significantly increased GM-CSF compared to the unstimulated control (*p* < 0.05) (Figure 6C). The anti-inflammatory cytokine IL-10 was significantly elevated by LPS, R848, Pam3CSK4, and PHA stimulation compared to the unstimulated control (*p* ≤ 0.0001) (Figure 6D).

At day 5, the levels of IL-17 were elevated in the LPS (*p* ≤ 0.0001), R848 (*p* < 0.05), Pam3CSK4 (*p* < 0.001), and PHA-stimulated and uninfected (*p* < 0.001) and infected (*p* ≤ 0.0001) conditions compared to the unstimulated infected control (Figure 6F). Similarly, IL-10 levels were elevated in the R848 (*p* < 0.001), Pam3CSK4 (*p* < 0.05), and PHA infected (*p* < 0.01) conditions compared to the unstimulated infected control (Figure 6H). Relevant mean ± SD for data depicted in Figure 6 are listed in Supplementary Table 5.

### TLR-Induced Inflammation Limits HIV Infection of CD4+ T Cells

**Figure 7:**
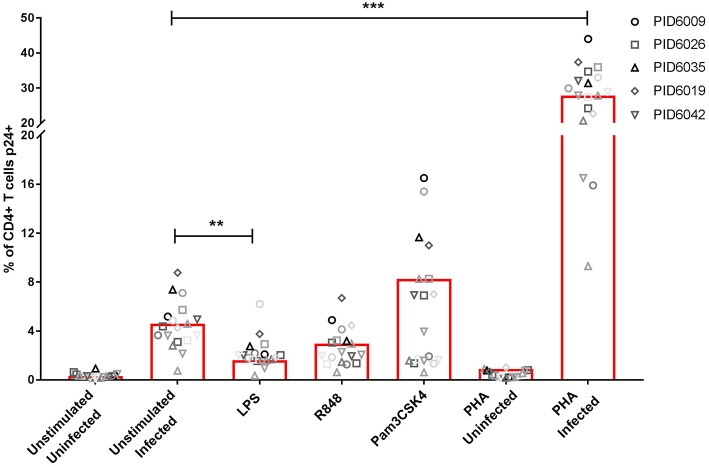
Infection rates (measured by p24 expression) of CD4+ T cells either unstimulated or stimulated with PHA or TLR agonists; LPS (TLR4), Pam3CSK4 (TLR1/2) or R848 (TLR7/8). Each symbol represents a donor, while different shades of each symbol represent repeats for that donor. PHA was used at a final concentration of 10 μg/ml. TLR agonists were used at a final concentration of 2 μg/ml. Significance was assessed by two-way ANOVA with Dunnett's multiple comparisons test. Significance is displayed as ^**^p < 0.01, ^***^p < 0.0001 compared to the unstimulated infected control. Sample size, *n* = 5, 4 donors run in quadruplicate, 1 donor run in duplicate.

We determined the effect of TLR-mediated inflammation on the susceptibility of CD4+ T cells to R5 tropic HIV infection with NL4-3 AD8 HIV. Stimulation with LPS (TLR4; *p* < 0.01), and R848 (TLR7/8) to a lesser extent, reduced HIV infection of CD4+ T cells compared to unstimulated cells (Figure 7). Pam3CSK4 induced infection similar to that of the unstimulated infected control. PHA-stimulation resulted in significantly more infection than all other conditions (*p* < 0.001), with approximately 25% of CD4+ T cells infected (Figure 7). Furthermore, using a combination of TLR agonist and PHA, we found that even in the presence of hyper activation, stimulation with either LPS or R848 protected CD4+ T cells from HIV infection (*p* > 0.0001; Supplementary Figure 12).

## Discussion

The purpose of this study was to identify the effects of TLR agonists on T cell activation, cytokine responses, and the ability of HIV to infect CD4+ T cells. TLR stimulation resulted in limited T cell activation, down-regulation of the CCR5 co-receptor necessary for HIV entry as well as potent inflammatory cytokine responses, creating an environment less conducive to HIV infection of CD4+ T cells.

CD4+ T cells have been shown to express various classes of TLRs (40), providing the ability to recognize and respond to TLR agonists. In our study, no significant increase in activation marker expression was observed for either CD4+ or CD8+ T cells when stimulated with agonists targeting TLR1/2, 7/8, or 4. It has been demonstrated that TLR receptors require co-stimulation, needing primary T-cell receptor (TCR) engagement to induce functional T cell responses (41). This is evident with the mitogen, PHA, where significant CD4+ T cell activation was observed following stimulation. PHA is a plant lectin that binds to carbohydrates on the cell surface, including the TCR, thereby inducing proliferation and activation of T lymphocytes (42–45). TLR7/8 agonist R848 induced subtle activation of CD4+ T cells, possibly due to intracellular recognition of R848 that may have stimulated an adaptive Th1 immune response even in the absence of TCR signaling (46). Effector CD8+ T cells are generally highly reliant on CD4+ T cell help for functional and memory responses (47). Thus, the modest activation of CD4+ T cells with R848 was also observed in the CD8+ T cells, providing further evidence of a cytotoxic Th1 response. Distinct activation of both CD4+ and CD8+ T cells was observed with PHA stimulation, likely due to the robust TCR engagement by PHA.

A distinct inflammatory response was observed when looking at the cytokine profiles induced by TLR stimulation compared to the unstimulated control. TLR agonists LPS and R848 elicited potent cytokine storms, similar to previous studies that showed increases in pro-inflammatory and chemotactic cytokines after stimulation with these TLR agonists (32–34). R848 also induced the strongest IFN-γ and IL-12p70 response, reminiscent of the anti-viral Th1 response (48). Furthermore, R848 stimulated the prolonged expression of IL-1α, IFN-γ, and TNF-α, further providing evidence of prolonged inflammatory responses associated with the adaptive immune response. Additionally, cytokines associated with immune modulation, such as IL-1RA, IL-4, and IL-10 were upregulated, presumably to prevent a prolonged inflammatory response. These data suggest that the inflammatory responses associated with LPS and R848 stimulation are regulated through immunomodulatory cytokines to counteract the exaggerated pro-inflammatory response. Furthermore, Pam3CSK4-mediated expression of IL-1RA, IL-4, and IL-10 was much lower than LPS or R848, suggesting that this TLR agonist induces either a Th2 biased or more regulatory cytokine response or a less potent Th1 response. This phenomenon may be in part due to this TLR1/2 agonist being analogous to gram-positive bacteria, which are generally less pathogenic and less inflammatory than gram-negative bacteria (49, 50).

The chemokine responses induced by TLR stimulation were potent, with prolonged IL-8, MIP-1α and MIP-1β expression. MIP-1α, MIP-1β, and RANTES are ligands for CCR5 and are associated with the recruitment of CCR5+ cells (51). In the female genital tract, these chemokines are important factors that are associated with increased risk for HIV acquisition in women (7, 8). Interestingly, MIP-1β was increased in the unstimulated and PHA infected conditions compared to their uninfected controls at day 5, suggesting that HIV itself induces the expression of MIP-1β. Dai and Stevenson (52) similarly showed that HIV-1 Nef induced the production of MIP-1β (52). Alternatively, the increased detection of soluble MIP-1β may be as a result of the competitive binding of HIV to CCR5 precluding MIP-1β binding. Furthermore, the increased expression of IL-8 and MCP-1 at day 3, which are chemotactic for neutrophils (53) and monocytes (54) respectively, provides the basis for the initiation of innate immune responses, which then further potentiate inflammation. Interestingly, higher prolonged IL-8 levels were observed with the bacterial TLR agonists LPS and Pam3CSK4 compared to the viral TLR agonist R848, suggesting that neutrophils would be sufficient for control and clearance of bacterial infections, whereas viral infections generally require a Th1 cytotoxic adaptive immune response to prevent infection. This is supported by the finding that IP-10 was induced at significantly higher concentrations by R848 than the bacterial TLR agonists. Compared to the unstimulated control at day 5, significantly less IP-10 was produced following LPS and R848 stimulation, but not by Pam3CSK4. This further supports the findings above which allude to the induction of an adaptive Th1 response to TLR7/8 activation, but not TLR1/2, while there is potential for the initiation of an adaptive response with continued TLR4 activation.

We found no effect of TLR stimulation on the production of GM-CSF, which was surprising given the inflammatory response observed. GM-CSF stimulates granulocyte and macrophage differentiation (55), which we postulated would be key in the innate immune response, especially against the bacterial TLR agonists. However, previous literature suggests that GM-CSF and TLRs appear to have a complex relationship. While GM-CSF stimulation is known to downregulate the expression of TLR1, 2 and 4 on human monocytes (56), it has also been shown to enhance LPS-mediated pro-inflammatory cytokine production in murine microglia via the upregulation of TLR4 and CD14 (57). Similarly, Bauer et al. (58) found that GM-CSF partially restored TLR-mediated functional responses of monocytes from septic patients (58). One possibility for blunted GM-CSF responses was that a PBMC model was used, so the need for GM-CSF may be lost in this lymphocyte-enriched system.

Th17 cells have an important role to play in the homeostasis and maintenance of the mucosal barrier (59–62), as well as increased susceptibility to HIV infection (63, 64). One of the limitations of this study is that we did not assess the Th17 cells by flow cytometry, however IL-17, as well as IL-7, are good surrogate indicators for Th17 cell functions (65–67). In our study, we found elevated IL-7 in all stimulation conditions, with the exception of Pam3CSK4, possibly suggesting a dampened or tolerogenic response to this TLR agonist. Furthermore, IL-17 was also elevated in all stimulated conditions at both day 3 and day 5. Interestingly, a less potent IL-17 response was seen at day 5 in R848 stimulated cells than those stimulated with either LPS or Pam3CSK4. These data suggest that the sensing of bacterial antigens, analogous to microbial translocation, may induce a prolonged and potent Th17 response to maintain homeostasis and integrity of the mucosal barrier. However, the observed significant dose-dependent increase of IL-17 with R848 stimulation suggests a stronger Th17 response with increased viral sensing.

Unexpectedly, TLR stimulation did not lead to increased HIV infection of CD4+ T cells. Initially, we assumed that the lack of CD4+ T cell activation could explain this observation, given that activated CD4+ T cells are preferentially and more easily infected (10, 68). However, in preliminary follow up experiments we found that even in a hyper activated setting, where PHA in addition to TLR agonists LPS or R848 were used, HIV infection rates of CD4+ T cells were still lower in the TLR agonist and PHA conditions compared to the PHA only control, thereby indicating other mechanisms at play. One possibility for the reduced HIV infection with LPS or R848 stimulation is through increased CC-binding chemokines which compete with HIV for CCR5 binding (69, 70), which was determined as a mechanism of resistance to R5-tropic viruses in elite controllers (71). This model system supports the concept of increased CC-binding chemokines relative to a decrease in CCR5 expression with a concomitant reduction in HIV infection. Furthermore, activation of TLR4 and TLR7/8, by LPS and R848 respectively, has been shown to induce type 1 interferons (72), which have potent antiviral effects and most likely played a role in the observed protection against HIV infection (73). Similarly, the observed protective effect suggests the induction of an innate antiviral response (74). This innate antiviral response likely involves host factors such as the APOBEC family of proteins, which are known to have nucleic acid editing functions (75–77), and SAMHD1, known to limit intracellular deoxynucleoside triphosphates thereby restricting viral replication (78, 79). This model of TLR stimulation creating an environment less conducive to HIV infection, is similar to the findings of inflammation and partial immune activation in highly exposed sero-negatives (HESN) (80, 81), in contrast to findings in other HESN cohorts showing immune quiescence (82). These data highlight the complex and heterogeneous nature of inflammation and immune activation, that determine HIV risk.

To further understand the effects of TLR stimulation on adaptive cellular activation, and address the limitations of this model system, these experiments could be repeated with the addition of a TCR stimulant such as anti-CD3/CD28 beads. Furthermore, we used markers of activation which are more relevant to assessing chronic immune activation (83). Markers of acute cellular activation, such as CD69 (83), may have been more appropriate. Additionally, innate antiviral pathways including interferon stimulated genes, type 1 interferons, APOBEC and SAMHD1 should be assessed to determine their potential roles in the observed protective effect by TLR agonists LPS and R848. Furthermore, the activation of innate immune cells such as monocytes and DCs were not assessed, and these could have provided valuable insight into the mechanisms of a TLR-mediated immunity. Antigen presenting cells such as DCs, macrophages and monocytes are generally the first line of defense in the recognition of pathogens, and subsequently activate the adaptive immune responses (84). However, monocytes constitute approximately 20% of the cell population in PBMCs (85), and so microbial recognition would have occurred. There was an overall decrease in cytokines from day 3 to day 5, which is likely due to the removal of stimulants at day 3 prior to HIV infection. In future, it will be important to assess HIV infection rates in the presence of continued TLR stimulation. Furthermore, we assessed cytokine expression from culture supernatants and could not distinguish the cellular origin of cytokines. As there was a lack of T cell activation in TLR-stimulated conditions, the observed cytokine responses were likely mediated by innate immune cells such as monocytes and neutrophils. Therefore, performing intracellular cytokine staining (ICS) for a few key cytokines would allow better discrimination of the main cells producing key inflammatory cytokines. Furthermore, ICS would allow better discrimination of cellular functionality, allowing clearer assessment of cellular subsets.

While PBMCs may not fully reflect cells in the genital tract of women, this culture system provides valuable insight into the mechanisms of TLR-induced inflammation. Additionally, PBMCs represent circulating cells which are a combination of peripheral and trafficked cells from the tissue, which better reflects an *in vivo* setting compared to depleted or purified immune cell models or cell lines. Jaspan et al. (12) previously reported that the extent of T cell activation in blood significantly predicted activation of these cells at the cervix (12). *Ex vivo* samples, such as cervical cells or explants are most biologically representative, however there are still many difficulties in obtaining and assessing immunity even in these types of samples (86–88).

These data highlight the inflammatory effects of TLR agonists on PBMCs, and the need for TCR engagement to induce activation of adaptive T cells. These results also provide insight into the nature of the immune responses elicited by various TLR agonists, with specific responses induced to particular pathogenic signals. Together, these data provide important mechanistic insights for HIV acquisition as the types of immune responses induced according to the pathogens or combinations of pathogens sensed, could govern HIV risk.

## Data Availability

The datasets generated for this study are available on request to the corresponding author.

## Ethics Statement

This study was carried out in accordance with the recommendations of the University of KwaZulu-Natal (UKZN) Biomedical Research Ethics Committee (BREC). All subjects gave written informed consent in accordance with the Declaration of Helsinki. The protocol was approved by the UKZN BREC (BE433/14).

## Author Contributions

SA, J-AP, LL, LM, and DA acquired funding for the study and edited the manuscript. LL, LM, J-AP, AS, and DA assisted in study design and analysis of data. RC designed and ran experimental procedures, acquired and analyzed data, wrote and edited the manuscript. AS assisted in study design, provided laboratory space for experimental procedures and edited the manuscript.

### Conflict of Interest Statement

The authors declare that the research was conducted in the absence of any commercial or financial relationships that could be construed as a potential conflict of interest.
