# Supplementary material for: Diminished HIV Infection of Target CD4+ T Cells in a Toll-Like Receptor 4 Stimulated in vitro Model
Source: Front Immunol. 2019 Jul 23;10:1705. doi: 10.3389/fimmu.2019.01705 (PMC6664077; doi:10.3389/fimmu.2019.01705)
Supplement: Supplementary file 1 [file Table_1.DOCX]

| Day 3 | CD38+HLA-DR+ | | CD38+HLA-DR- | | CD38-HLA-DR+ | | CD38-HLA-DR- | |
| --- | --- | --- | --- | --- | --- | --- | --- | --- |
|  | mean | SD | mean | SD | mean | SD | mean | SD |
| Unstimulated | 1.96 | 1.35 | 20.43 | 9.44 | 8.05 | 4.19 | 69.58 | 12.84 |
| LPS | 2.35 | 1.68 | 18.93 | 8.50 | 9.14 | 4.56 | 69.57 | 12.77 |
| R848 | 3.08 | 1.90 | 21.80 | 8.79 | 8.22 | 4.34 | 66.93 | 12.37 |
| Pam3CSK4 | 2.30 | 1.68 | 18.00 | 7.26 | 10.16 | 5.22 | 69.57 | 11.99 |
| PHA | 26.38 | 12.82 | 26.79 | 9.21 | 13.33 | 3.90 | 33.53 | 16.81 |
|  |  |  |  |  |  |  |  |  |
| Day 5 | CD38+HLA-DR+ | | CD38+HLA-DR- | | CD38-HLA-DR+ | | CD38-HLA-DR- | |
|  | mean | SD | mean | SD | mean | SD | mean | SD |
| Unstimulated Uninfected | 2.57 | 2.81 | 18.01 | 9.67 | 10.47 | 8.39 | 68.95 | 14.15 |
| Unstimulated Infected | 3.07 | 2.81 | 19.42 | 10.71 | 11.08 | 8.40 | 66.46 | 13.50 |
| LPS | 3.07 | 3.17 | 17.88 | 9.53 | 11.19 | 8.51 | 67.86 | 15.17 |
| R848 | 4.12 | 3.65 | 21.82 | 10.87 | 8.89 | 7.24 | 65.17 | 14.56 |
| Pam3CSK4 | 3.17 | 2.69 | 17.18 | 8.95 | 12.33 | 8.27 | 67.32 | 13.95 |
| PHA Uninfected | 26.27 | 14.85 | 45.12 | 8.79 | 6.63 | 3.01 | 22.00 | 11.55 |
| PHA Infected | 24.96 | 14.60 | 45.72 | 8.38 | 5.95 | 2.49 | 23.37 | 9.96 |

Supplementary Table 1: Mean percentage (%) and standard deviations (SD) of CD4+ T cells expressing cellular activation markers CD38 and HLA-DR in unstimulated or stimulated (LPS, R848, Pam3CSK4 and PHA) conditions at day 3 (top) and day 5 (bottom). Sample size, n=5, 4 donors run in quadruplicate, 1 donor run in duplicate.
