# Supplementary material for: Diminished HIV Infection of Target CD4+ T Cells in a Toll-Like Receptor 4 Stimulated in vitro Model
Source: Front Immunol. 2019 Jul 23;10:1705. doi: 10.3389/fimmu.2019.01705 (PMC6664077; doi:10.3389/fimmu.2019.01705)
Supplement: Supplementary file 2 [file Table_2.DOCX]

| Day 3 | CD38+HLA-DR+ | | CD38+HLA-DR- | | CD38-HLA-DR+ | | CD38-HLA-DR- | |
| --- | --- | --- | --- | --- | --- | --- | --- | --- |
|  | mean | SD | mean | SD | mean | SD | mean | SD |
| Unstimulated | 1.28 | 0.91 | 8.44 | 6.40 | 9.19 | 6.07 | 81.09 | 10.47 |
| LPS | 2.20 | 2.23 | 8.51 | 5.27 | 10.79 | 6.98 | 78.50 | 11.38 |
| R848 | 4.13 | 3.41 | 11.83 | 6.40 | 11.06 | 6.84 | 73.01 | 11.82 |
| Pam3CSK4 | 1.25 | 0.88 | 7.29 | 4.47 | 10.19 | 6.50 | 81.26 | 9.37 |
| PHA | 30.37 | 14.18 | 15.50 | 6.93 | 17.71 | 5.27 | 36.43 | 17.87 |
|  |  |  |  |  |  |  |  |  |
| Day 5 | CD38+HLA-DR+ | | CD38+HLA-DR- | | CD38-HLA-DR+ | | CD38-HLA-DR- | |
|  | mean | SD | mean | SD | mean | SD | mean | SD |
| Unstimulated Uninfected | 1.69 | 1.61 | 6.07 | 6.49 | 13.39 | 12.49 | 78.84 | 15.75 |
| Unstimulated Infected | 3.29 | 3.45 | 8.85 | 7.98 | 13.48 | 10.97 | 74.36 | 15.27 |
| LPS | 2.66 | 2.57 | 7.32 | 6.37 | 14.37 | 13.30 | 75.67 | 16.62 |
| R848 | 7.40 | 5.24 | 14.24 | 8.34 | 12.92 | 11.43 | 65.46 | 15.66 |
| Pam3CSK4 | 2.38 | 1.87 | 6.66 | 5.68 | 13.84 | 11.53 | 77.11 | 13.69 |
| PHA Uninfected | 38.35 | 16.79 | 41.41 | 13.43 | 4.08 | 2.53 | 16.14 | 9.15 |
| PHA Infected | 38.91 | 16.31 | 41.87 | 13.32 | 3.79 | 2.29 | 15.45 | 9.89 |

Supplementary Table 2: Mean percentage (%) and standard deviations (SD) of CD8+ T cells expressing cellular activation markers CD38 and HLA-DR in unstimulated or stimulated (LPS, R848, Pam3CSK4 and PHA) conditions at day 3 (top) and day 5 (bottom). Sample size, n=5, 4 donors run in quadruplicate, 1 donor run in duplicate.
