# Supplementary material for: Diminished HIV Infection of Target CD4+ T Cells in a Toll-Like Receptor 4 Stimulated in vitro Model
Source: Front Immunol. 2019 Jul 23;10:1705. doi: 10.3389/fimmu.2019.01705 (PMC6664077; doi:10.3389/fimmu.2019.01705)
Supplement: Supplementary file 3 [file Table_3.DOCX]

| Pro-inflammatory cytokines Day 3 | IL-1α | | IL-1β | | IL-6 | | IL-12p70 | | IFN-γ | | TNF-α | |
| --- | --- | --- | --- | --- | --- | --- | --- | --- | --- | --- | --- | --- |
|  | mean | SD | mean | SD | mean | SD | mean | SD | mean | SD | mean | SD |
| Unstimulated | 0.900 | 0.713 | 0.503 | 1.111 | 1.795 | 0.711 | 0.701 | 0.582 | 1.738 | 1.029 | 1.792 | 0.580 |
| LPS | 2.347 | 0.404 | 3.170 | 0.306 | 4.229 | 0.597 | 1.482 | 0.175 | 2.722 | 0.136 | 3.222 | 0.655 |
| R848 | 2.441 | 0.321 | 3.570 | 0.418 | 4.216 | 0.460 | 1.771 | 0.246 | 3.006 | 0.273 | 3.965 | 0.649 |
| Pam3CSK4 | 1.573 | 0.366 | 2.357 | 0.249 | 3.935 | 0.401 | 1.212 | 0.260 | 2.533 | 0.194 | 2.828 | 0.558 |
| PHA | 2.506 | 0.348 | 3.289 | 0.202 | 4.279 | 0.602 | 1.534 | 0.163 | 3.207 | 0.309 | 3.456 | 0.643 |
|  |  |  |  |  |  |  |  |  |  |  |  |  |
| Pro-inflammatory cytokines Day 5 | IL-1α | | IL-1β | | IL-6 | | IL-12p70 | | IFN-γ | | TNF-α | |
|  | mean | SD | mean | SD | mean | SD | mean | SD | mean | SD | mean | SD |
| Unstimulated Uninfected | 0.943 | 1.158 | 0.505 | 0.371 | 2.091 | 0.464 | 0.845 | 0.546 | 1.572 | 1.238 | 2.140 | 0.456 |
| Unstimulated Infected | 0.966 | 0.904 | 0.569 | 0.463 | 2.071 | 0.575 | 0.849 | 0.700 | 1.954 | 0.810 | 1.993 | 0.328 |
| LPS | 1.426 | 0.504 | 1.731 | 0.401 | 3.167 | 0.742 | 1.087 | 0.563 | 2.368 | 0.293 | 2.185 | 0.225 |
| R848 | 1.633 | 0.213 | 2.070 | 0.268 | 3.107 | 0.389 | 1.208 | 0.302 | 2.488 | 0.197 | 2.403 | 0.607 |
| Pam3CSK4 | 1.318 | 0.569 | 1.286 | 0.245 | 3.351 | 0.464 | 1.036 | 0.539 | 2.421 | 0.308 | 2.296 | 0.216 |
| PHA Uninfected | 1.546 | 0.347 | 1.780 | 0.246 | 3.031 | 0.567 | 0.900 | 0.428 | 2.319 | 0.287 | 2.082 | 0.499 |
| PHA Infected | 1.498 | 0.288 | 1.774 | 0.456 | 3.020 | 0.666 | 1.018 | 0.546 | 2.366 | 0.255 | 2.212 | 0.338 |

Supplementary Table 3: Mean concentrations (Log_10_ pg/ml) and standard deviations (SD) of pro-inflammatory cytokines in cell culture supernatants at day 3 (top) and day 5 (bottom) from unstimulated, TLR or PHA stimulated PBMCs. Sample size, n=5, 4 donors run in quadruplicate, 1 donor run in duplicate.

Supplementary Table 3: Mean concentrations (Log_10_ pg/ml) and standard deviations (SD) of pro-inflammatory cytokines in cell culture supernatants at day 3 (top) and day 5 (bottom) from unstimulated, TLR or PHA stimulated PBMCs. Sample size, n=5, 4 donors run in quadruplicate, 1 donor run in duplicate.
