# Supplementary material for: Diminished HIV Infection of Target CD4+ T Cells in a Toll-Like Receptor 4 Stimulated in vitro Model
Source: Front Immunol. 2019 Jul 23;10:1705. doi: 10.3389/fimmu.2019.01705 (PMC6664077; doi:10.3389/fimmu.2019.01705)
Supplement: Supplementary file 4 [file Table_4.DOCX]

| Chemokines Day 3 | IL-8 | | MIP-1α | | MIP-1β | | IP-10 | | MCP-1 | | RANTES | |
| --- | --- | --- | --- | --- | --- | --- | --- | --- | --- | --- | --- | --- |
|  | mean | SD | mean | SD | mean | SD | mean | SD | mean | SD | mean | SD |
| Unstimulated | 3.551 | 0.632 | 0.950 | 0.820 | 2.052 | 0.479 | 2.653 | 0.958 | 3.217 | 0.833 | 2.275 | 0.683 |
| LPS | 4.787 | 0.175 | 3.751 | 0.325 | 3.951 | 0.338 | 2.374 | 0.481 | 3.693 | 0.344 | 3.046 | 0.716 |
| R848 | 4.720 | 0.284 | 3.751 | 0.330 | 4.082 | 0.244 | 3.228 | 0.933 | 3.693 | 0.301 | 3.063 | 0.644 |
| Pam3CSK4 | 4.833 | 0.339 | 3.573 | 0.278 | 3.696 | 0.352 | 2.456 | 0.668 | 3.781 | 0.387 | 2.844 | 0.665 |
| PHA | 4.946 | 0.395 | 3.762 | 0.327 | 4.290 | 0.360 | 3.507 | 0.713 | 3.704 | 0.327 | 3.428 | 0.631 |
|  |  |  |  |  |  |  |  |  |  |  |  |  |
| Chemokines Day 5 | IL-8 | | MIP-1α | | MIP-1β | | IP-10 | | MCP-1 | | RANTES | |
|  | mean | SD | mean | SD | mean | SD | mean | SD | mean | SD | mean | SD |
| Unstimulated Uninfected | 3.781 | 0.244 | 1.393 | 0.648 | 1.594 | 0.281 | 3.093 | 1.046 | 3.514 | 0.404 | 2.040 | 0.522 |
| Unstimulated Infected | 3.697 | 0.380 | 1.372 | 0.644 | 2.387 | 0.613 | 3.426 | 1.186 | 3.649 | 0.303 | 2.002 | 0.527 |
| LPS | 4.534 | 0.325 | 2.484 | 0.784 | 2.948 | 0.667 | 2.531 | 0.638 | 3.713 | 0.238 | 2.215 | 0.395 |
| R848 | 4.292 | 0.194 | 2.909 | 0.498 | 3.159 | 0.285 | 2.675 | 0.716 | 3.608 | 0.160 | 2.446 | 0.242 |
| Pam3CSK4 | 4.700 | 0.196 | 2.599 | 0.486 | 3.056 | 0.238 | 2.906 | 0.943 | 3.832 | 0.270 | 2.115 | 0.496 |
| PHA Uninfected | 4.415 | 0.151 | 2.255 | 0.589 | 2.090 | 0.752 | 2.466 | 0.535 | 3.583 | 0.316 | 2.393 | 0.584 |
| PHA Infected | 4.408 | 0.307 | 2.354 | 0.521 | 2.933 | 0.294 | 2.742 | 0.765 | 3.582 | 0.260 | 2.526 | 0.612 |

Supplementary Table 4: Mean concentrations (Log_10_ pg/ml) and standard deviations (SD) of chemotactic cytokines in cell culture supernatants at day 3 (top) and day 5 (bottom) from unstimulated, TLR or PHA stimulated PBMCs. Sample size, n=5, 4 donors run in quadruplicate, 1 donor run in duplicate.

Supplementary Table 3: Mean concentrations (Log_10_ pg/ml) and standard deviations (SD) of pro-inflammatory cytokines in cell culture supernatants at day 3 (top) and day 5 (bottom) from unstimulated, TLR or PHA stimulated PBMCs. Sample size, n=5, 4 donors run in quadruplicate, 1 donor run in duplicate.
