# Supplementary material for: Diminished HIV Infection of Target CD4+ T Cells in a Toll-Like Receptor 4 Stimulated in vitro Model
Source: Front Immunol. 2019 Jul 23;10:1705. doi: 10.3389/fimmu.2019.01705 (PMC6664077; doi:10.3389/fimmu.2019.01705)
Supplement: Supplementary file 5 [file Table_5.DOCX]

| Haematopoietic and anti-inflammatory cytokines Day 3 | IL-7 | | IL-17 | | GM-CSF | | IL-10 | |
| --- | --- | --- | --- | --- | --- | --- | --- | --- |
|  | mean | SD | mean | SD | mean | SD | mean | SD |
| Unstimulated | -0.193 | 0.997 | 1.772 | 0.297 | 1.819 | 0.870 | 1.219 | 0.330 |
| LPS | 0.599 | 0.616 | 2.588 | 0.108 | 2.178 | 0.572 | 2.771 | 0.240 |
| R848 | 0.618 | 0.617 | 2.584 | 0.110 | 2.186 | 0.516 | 2.865 | 0.249 |
| Pam3CSK4 | 0.189 | 0.812 | 2.472 | 0.205 | 2.142 | 0.601 | 2.140 | 0.212 |
| PHA | 0.789 | 0.652 | 3.332 | 0.333 | 2.421 | 0.389 | 3.137 | 0.151 |
|  |  |  |  |  |  |  |  |  |
| Haematopoietic and anti-inflammatory cytokines Day 5 | IL-7 | | IL-17 | | GM-CSF | | IL-10 | |
|  | mean | SD | mean | SD | mean | SD | mean | SD |
| Unstimulated Uninfected | -0.206 | 1.129 | 1.879 | 0.411 | 1.793 | 0.995 | 1.372 | 0.292 |
| Unstimulated Infected | -0.040 | 0.963 | 1.887 | 0.433 | 1.800 | 0.930 | 1.373 | 0.285 |
| LPS | 0.005 | 0.819 | 2.509 | 0.511 | 1.980 | 0.795 | 1.494 | 0.292 |
| R848 | 0.203 | 0.928 | 2.307 | 0.191 | 2.046 | 0.660 | 1.729 | 0.197 |
| Pam3CSK4 | -0.253 | 0.967 | 2.496 | 0.472 | 2.069 | 0.713 | 1.599 | 0.139 |
| PHA Uninfected | -0.597 | 1.138 | 2.389 | 0.295 | 1.891 | 0.785 | 1.557 | 0.164 |
| PHA Infected | 0.317 | 0.643 | 2.514 | 0.491 | 1.910 | 0.824 | 1.636 | 0.273 |

Supplementary Table 5: Mean concentrations (Log_10_ pg/ml) and standard deviations (SD) of haematopoietic and anti-inflammatory cytokines in cell culture supernatants at day 3 (top) and day 5 (bottom) from unstimulated, TLR or PHA stimulated PBMCs. Sample size, n=5, 4 donors run in quadruplicate, 1 donor run in duplicate.

Supplementary Table 3: Mean concentrations (Log_10_ pg/ml) and standard deviations (SD) of pro-inflammatory cytokines in cell culture supernatants at day 3 (top) and day 5 (bottom) from unstimulated, TLR or PHA stimulated PBMCs. Sample size, n=5, 4 donors run in quadruplicate, 1 donor run in duplicate.
