# Supplementary figures and images for: Diminished HIV Infection of Target CD4+ T Cells in a Toll-Like Receptor 4 Stimulated in vitro Model
Source: Front Immunol. 2019 Jul 23;10:1705. doi: 10.3389/fimmu.2019.01705 (PMC6664077; doi:10.3389/fimmu.2019.01705)

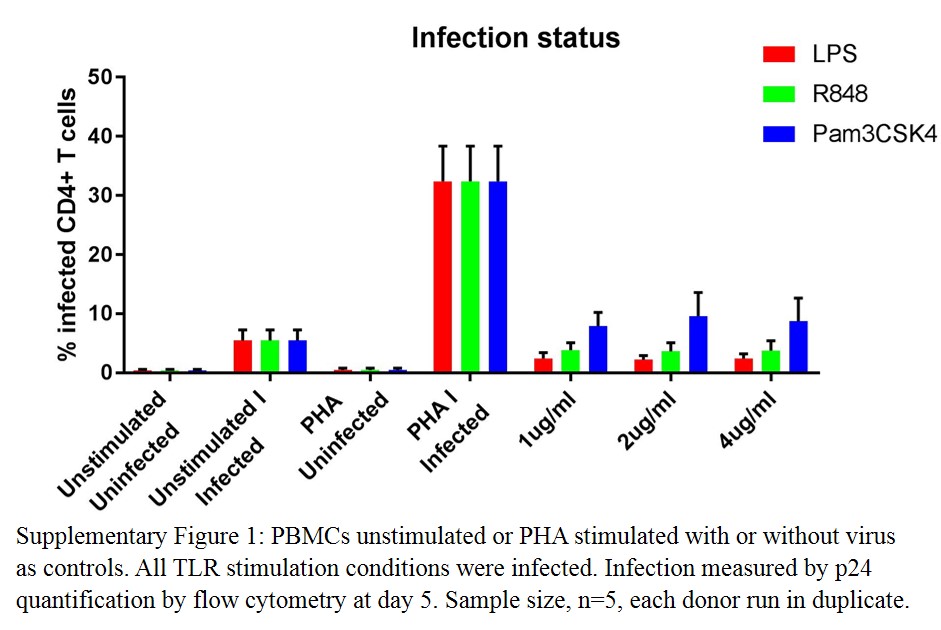

Supplement: Supplementary file 6 [file Image_1.JPEG]

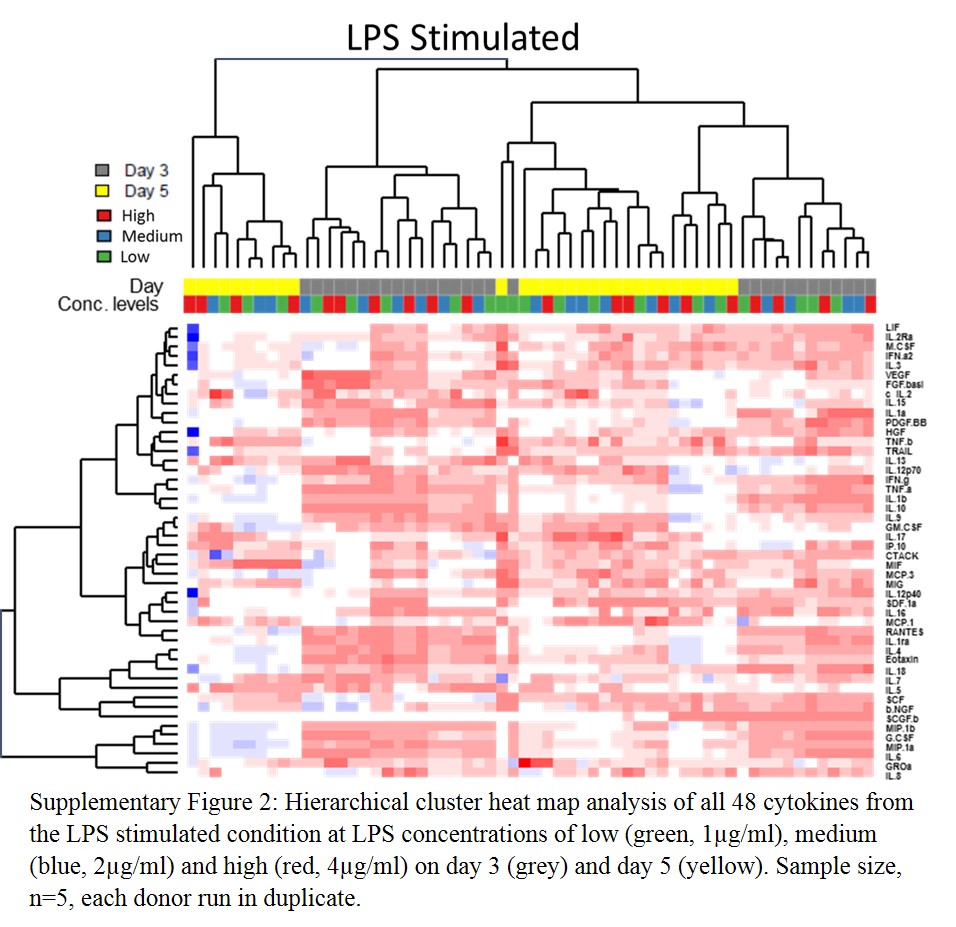

Supplement: Supplementary file 7 [file Image_2.JPEG]

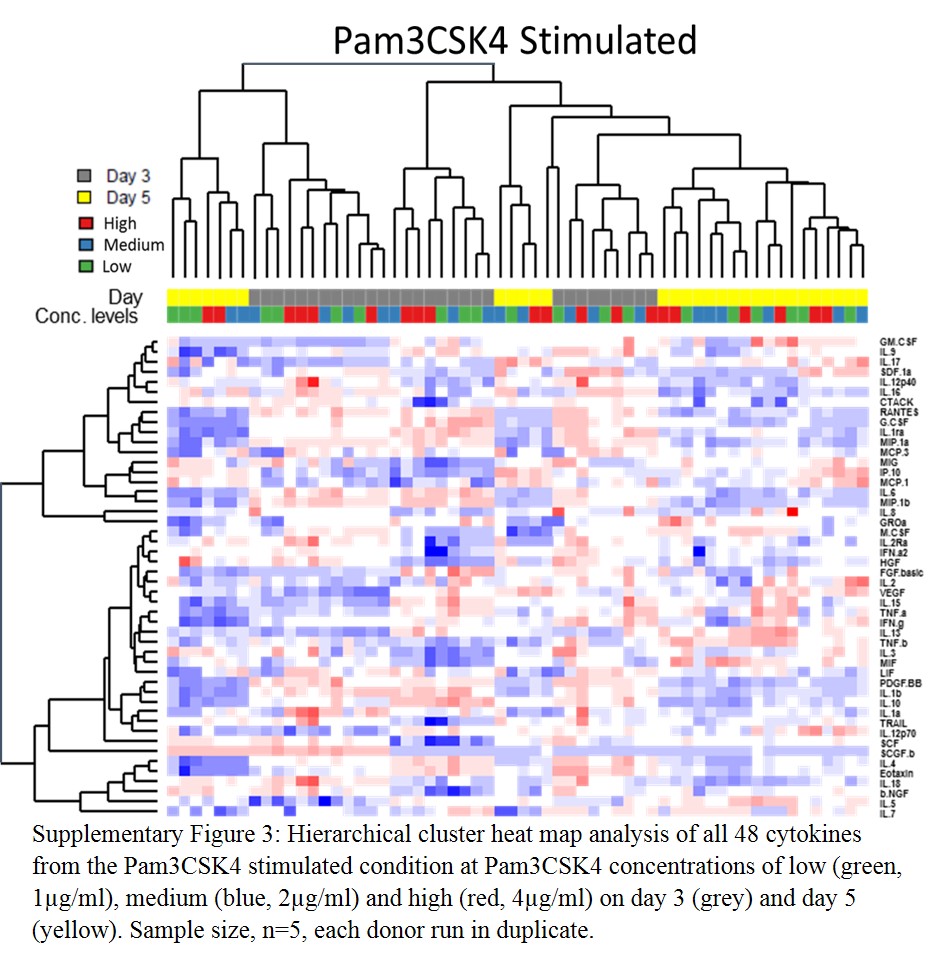

Supplement: Supplementary file 8 [file Image_3.JPEG]

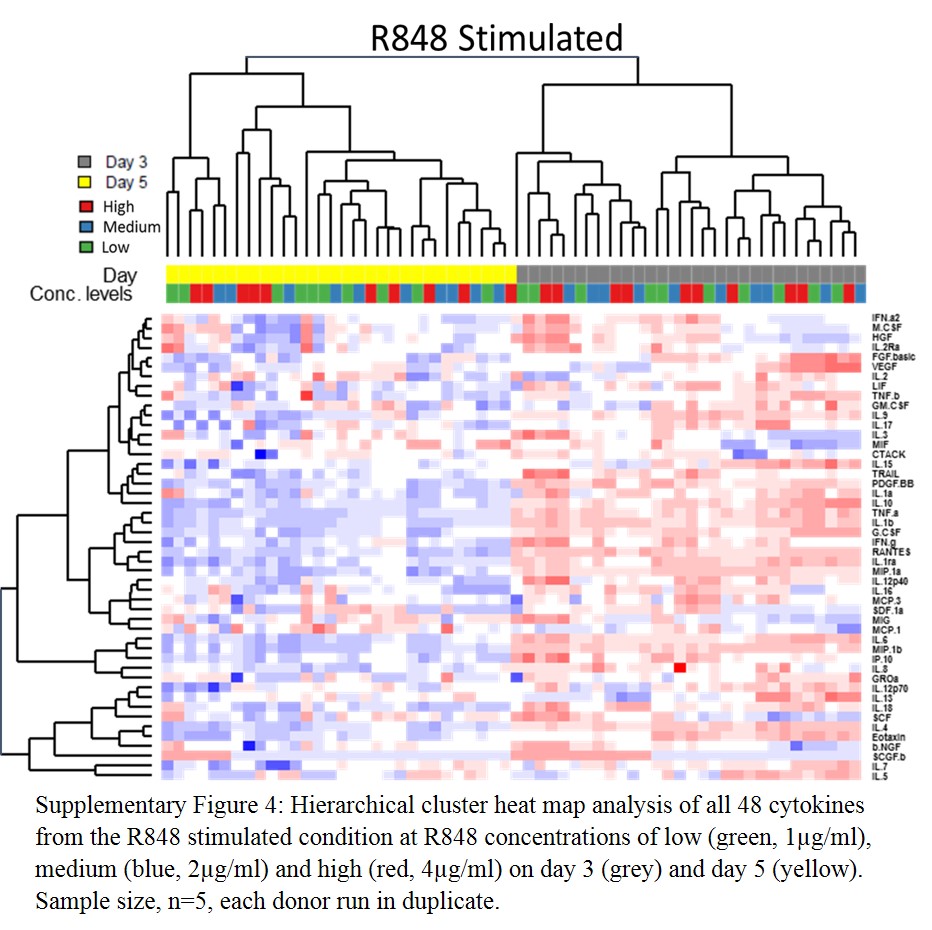

Supplement: Supplementary file 9 [file Image_4.JPEG]

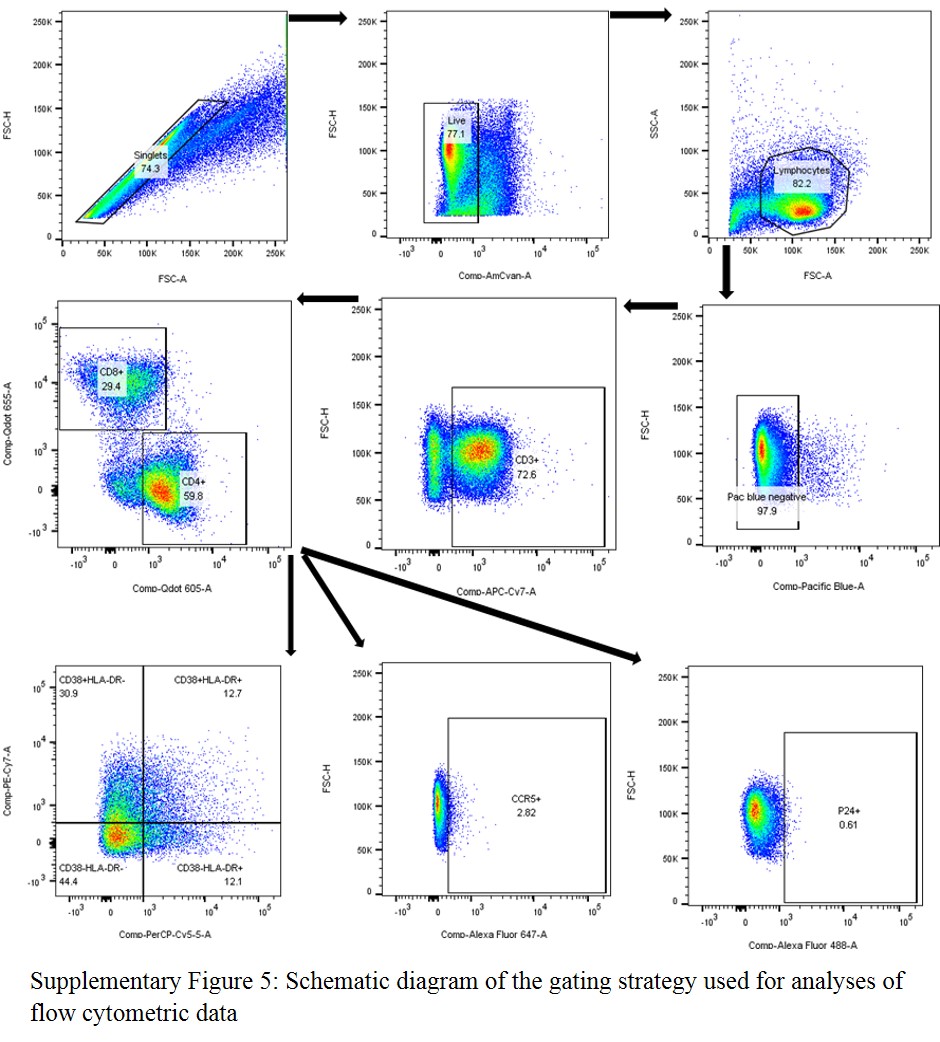

Supplement: Supplementary file 10 [file Image_5.JPEG]

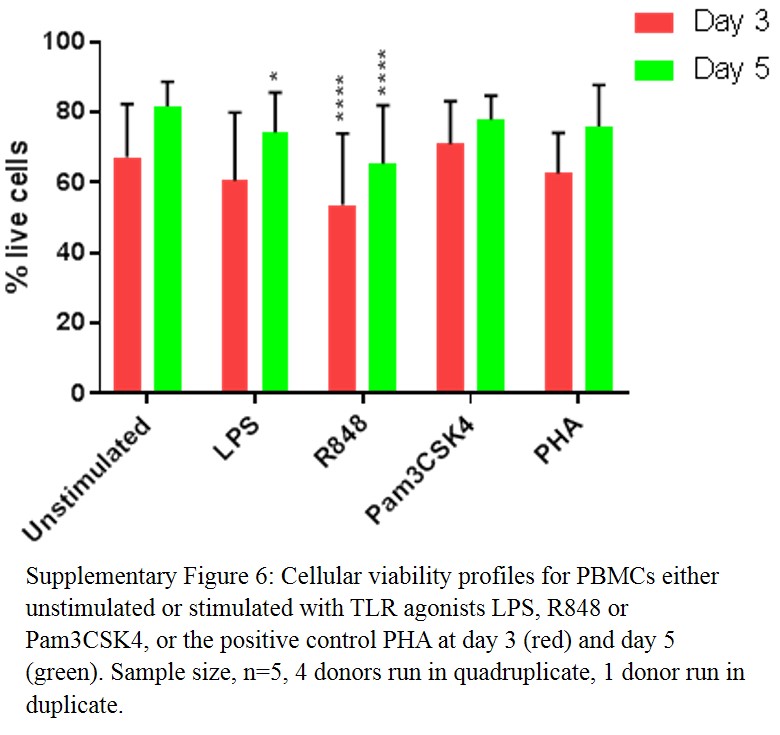

Supplement: Supplementary file 11 [file Image_6.JPEG]

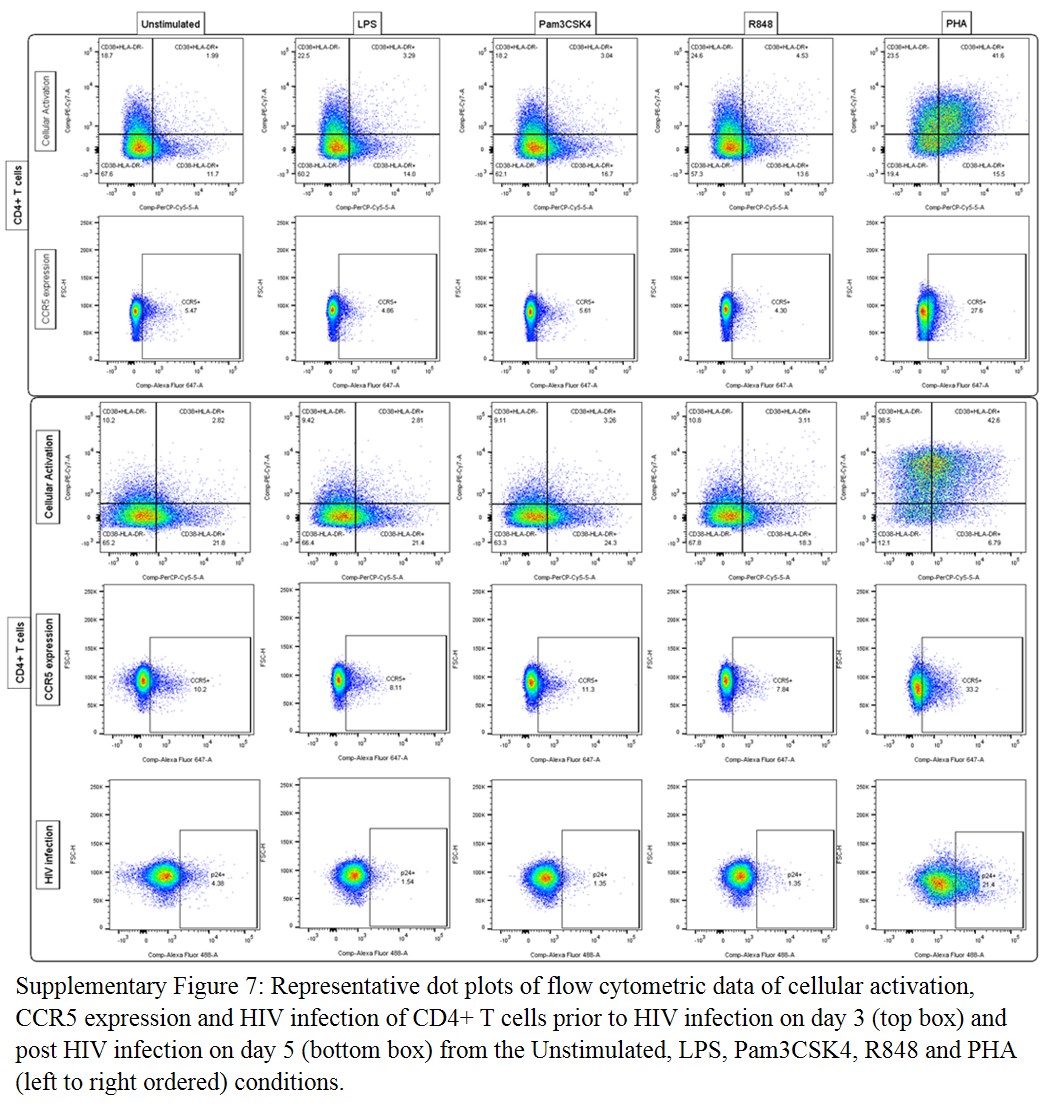

Supplement: Supplementary file 12 [file Image_7.jpg]

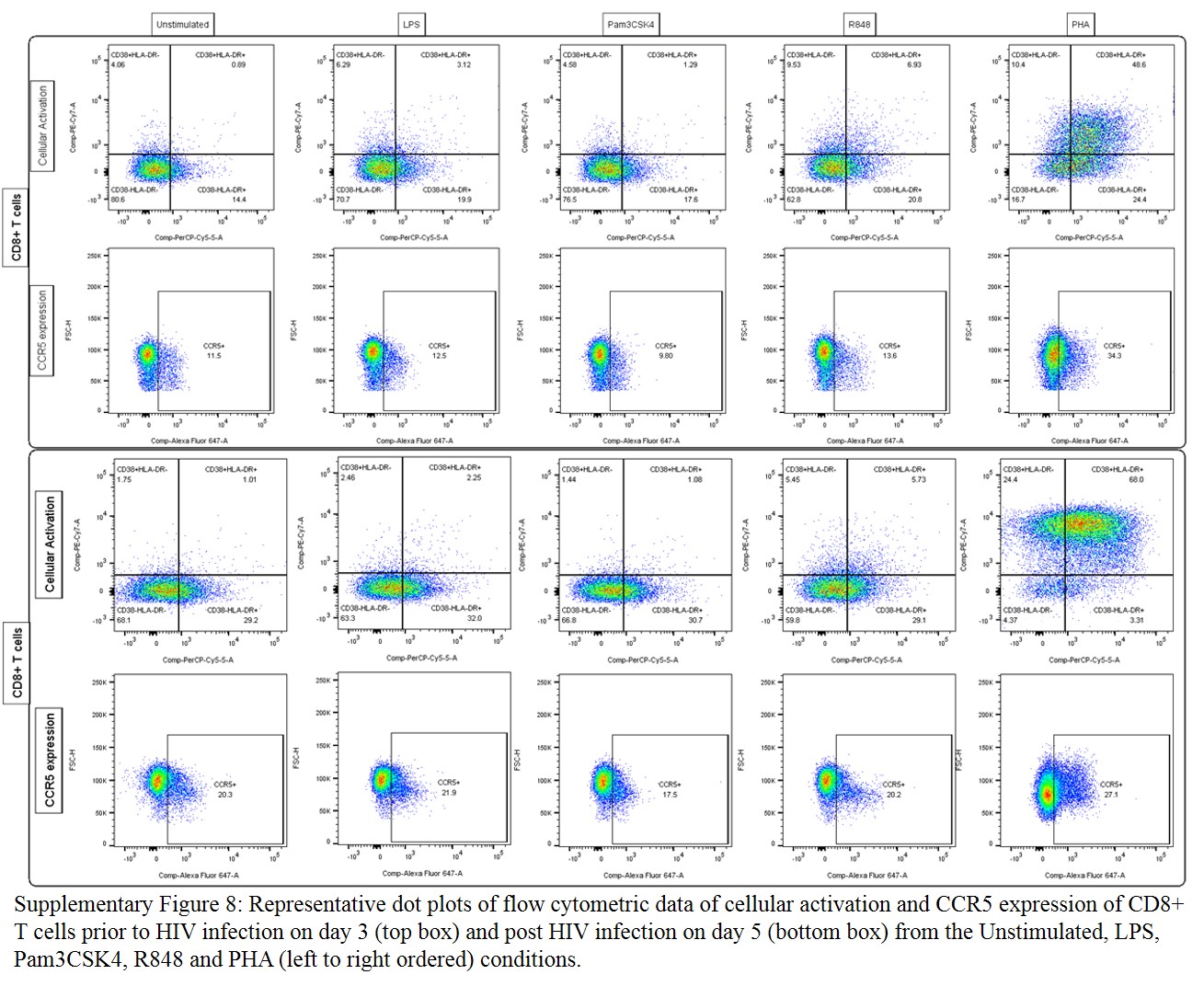

Supplement: Supplementary file 13 [file Image_8.jpg]

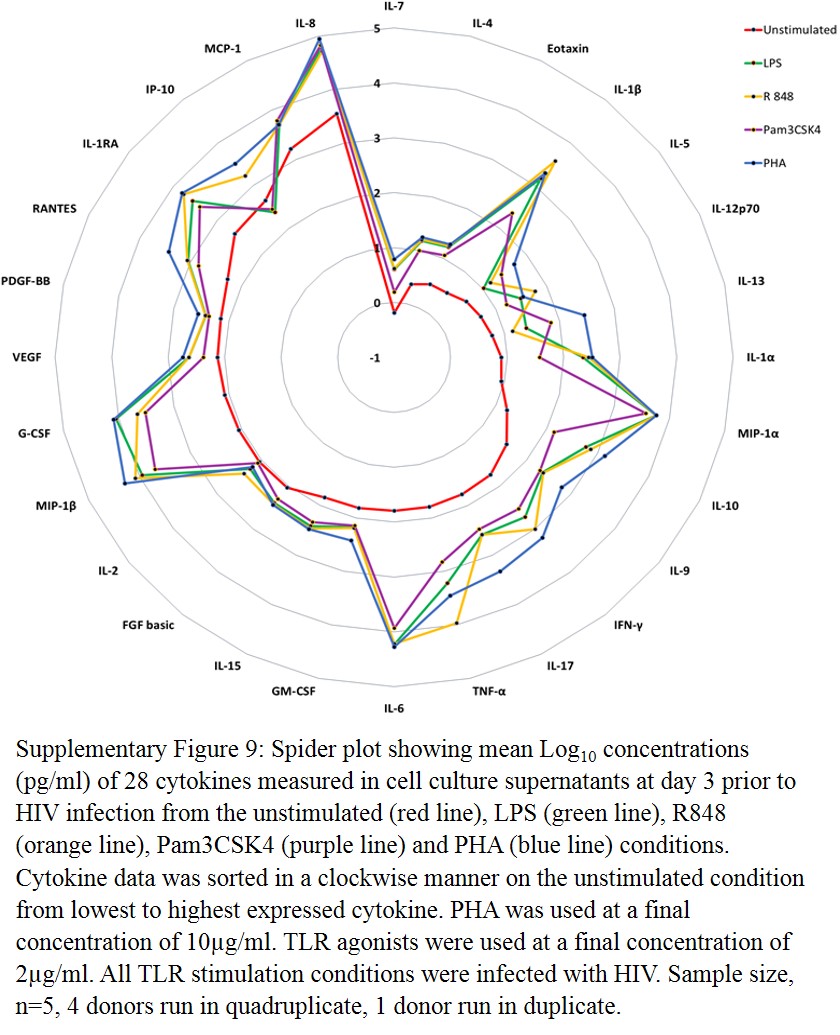

Supplement: Supplementary file 14 [file Image_9.jpg]

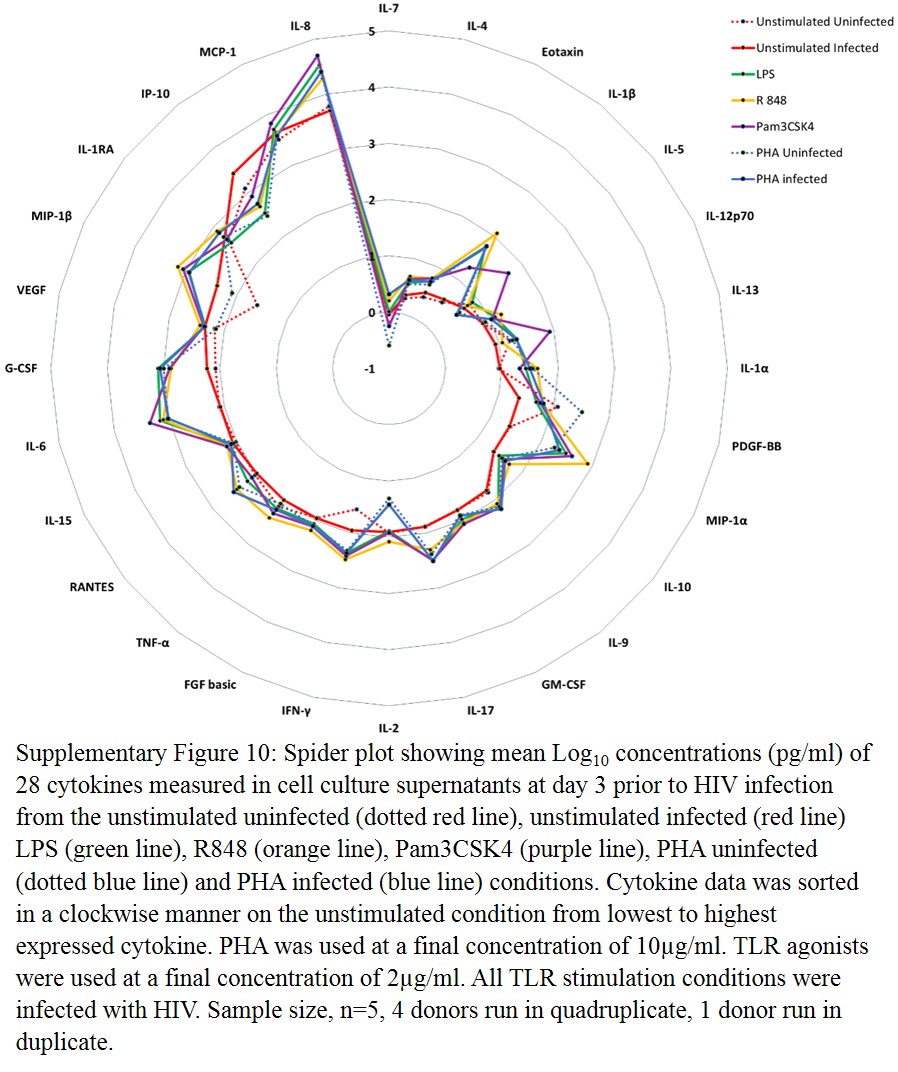

Supplement: Supplementary file 15 [file Image_10.jpg]

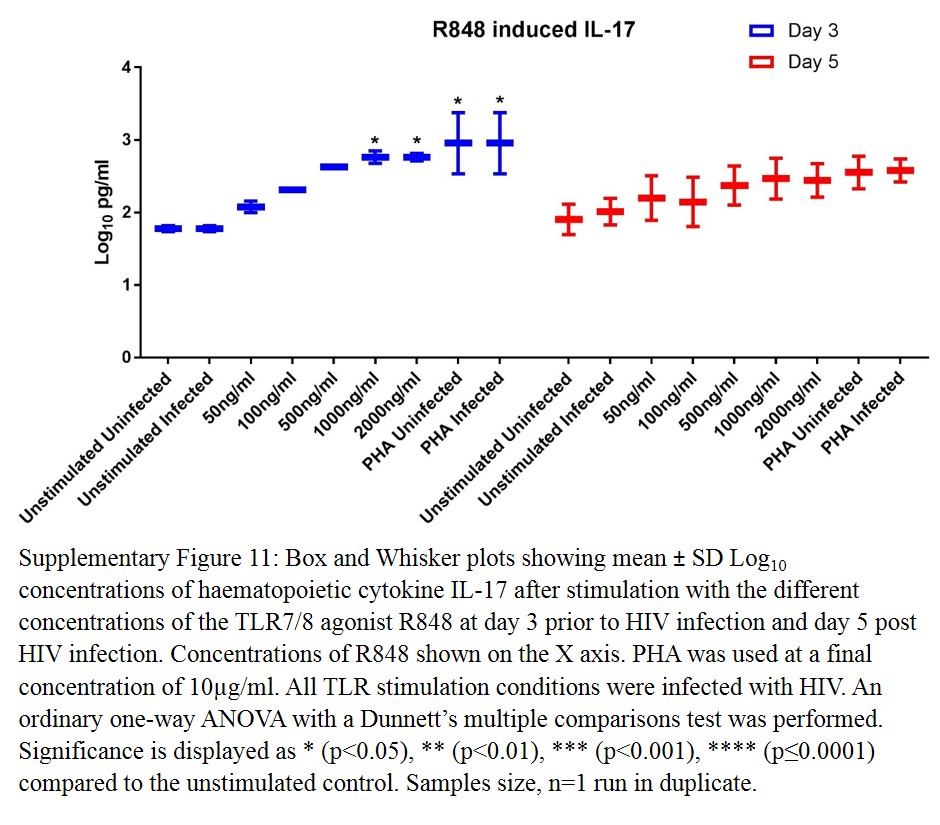

Supplement: Supplementary file 16 [file Image_11.jpg]

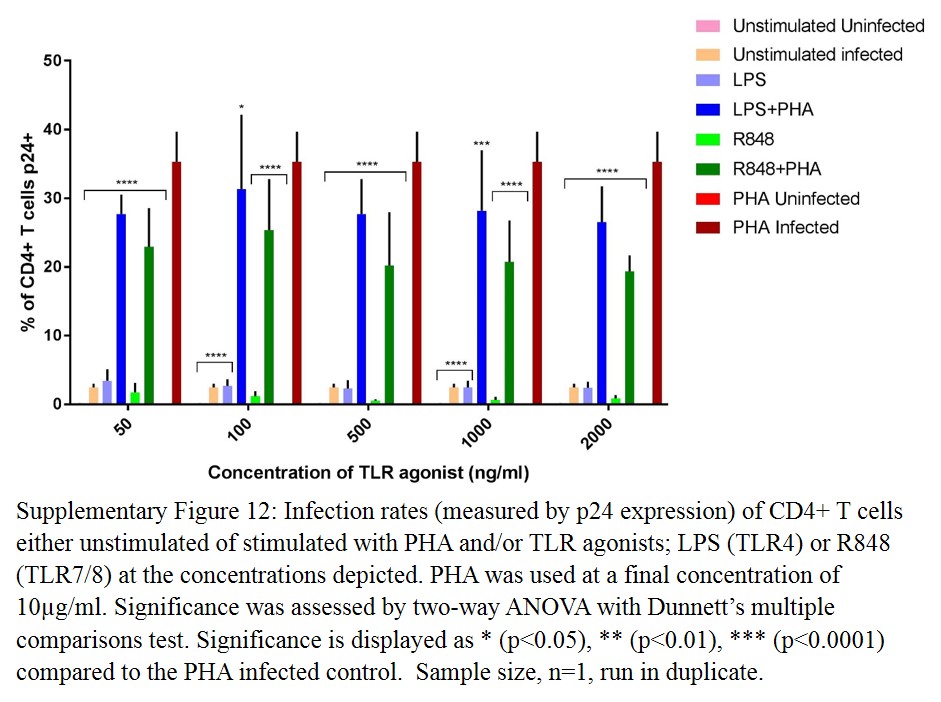

Supplement: Supplementary file 17 [file Image_12.jpg]
